# 3LPS-binding protein and its interactions with *P*. *gingivalis* LPS modulate pro-inflammatory response and Toll-like receptor signaling in human oral keratinocytes

**DOI:** 10.1371/journal.pone.0173223

**Published:** 2017-04-06

**Authors:** Pei-Hui Ding, Richard P. Darveau, Cun-Yu Wang, Lijian Jin

**Affiliations:** 1 Department of Periodontology, The Second Affiliated Hospital of Zhejiang University School of Medicine, Hangzhou, China; 2 Discipline of Periodontology, Faculty of Dentistry, The University of Hong Kong, Hong Kong SAR, China; 3 Department of Periodontics, University of Washington School of Dentistry, D-570 Health Sciences Building, Seattle, WA, United States of America; 4 Department of Oral Biology & Medicine, University of California Los Angeles School of Dentistry, CHS, Room 33–030, Los Angeles, CA, United States of America; University of the Pacific, UNITED STATES

## Abstract

Lipopolysaccharide (LPS)-binding protein (LBP) as an acute-phase protein plays a crucial role in innate host response to bacterial challenge. Our previous study shows that LBP expression in human gingiva is associated with periodontal status. *Porphyromonas gingivalis* is a keystone periodontopathogen, and its LPS with lipid A structural heterogeneity critically accounts for periodontal pathogenesis. This study investigated the effects of LBP and its interactions with two featured isoforms of *P*. *gingivalis* LPS (tetra-acylated LPS_1435/1449_ and penta-acylated LPS_1690_) on the expression of pro-inflammatory cytokines in human oral keratinocytes (HOKs), and the involvement of Toll-like receptor (TLR) signaling. HOKs were pre-incubated with recombinant human LBP (rhLBP) at 10ng/ml, 100ng/ml and 1μg/ml for 1 h, followed by the treatment of *P*. *gingivalis* LPS_1690_ or LPS_1435/1449_ for 3h or 24h respectively. The expression of IL-6 and IL-8, and involvements of TLR2 and TLR4 were analyzed. The genes associated with TLR signaling were assessed by PCR array. Interestingly, rhLBP *per se* significantly up-regulated the expression of IL-6 and IL-8 in HOKs (*p*<0.05), which was blocked by TLR2 antibody (*p*<0.001). LPS_1435/1449_ down-regulated more significantly rhLBP-induced IL-6 and IL-8 mRNAs with reference to *P*. *gingivalis* LPS_1690_ (approximately 80% vs. 40%, *p*<0.05; and 90% vs. 36%, *p*<0.001, respectively). Moreover, rhLBP markedly down-regulated the gene expression of TLRs and their adaptors such as CD180 (-2.44 folds) and MD-1 (-9.62 folds), while the interaction of *P*. *gingivalis* LPS_1435/1449_ with rhLBP greatly up-regulated both transcripts (7.11 and 4.05 folds, respectively). Notably, *P*. *gingivalis* LPS_1690_-rhLBP interaction dramatically up-regulated CD180 transcript (20.86 folds) and significantly down-regulated MD-1 transcript (-6.93 folds). This pioneering study shows that rhLBP enables to enhance the expression of pro-inflammatory cytokines in HOKs through TLR2 signaling pathway. *P*. *gingivalis* LPS with different lipid A structures down-regulates to different extents rhLBP-induced cytokine expression, possibly through fine-tuning of the CD180-MD1 complex and relevant TLRs.

## Introduction

Lipopolysaccharide (LPS)-binding protein (LBP) as an acute-phase protein is primarily produced by hepatocytes [1]. It regulates the property of LPS and modulates innate host responses to bacterial challenge [2]. LBP has a classical dual role, namely enhancing LPS-induced cellular activation at a low concentration and neutralizing the effects of bacterial endotoxins at a high concentration [3,4]. Additionally, it could interact with bacteria and other bacterial components [5–7]. In addition to hepatocytes, LBP could be synthesized by intestinal epithelial cells [8] and respiratory type II epithelial cells [9]. It is worthy to note that our early study shows that human gingival epithelia can produce LBP with a well-lined expression at the dentogingival niche, and its expression level in periodontally healthy subjects is significantly higher than that in chronic periodontitis patients. These findings suggest that LBP may be significantly involved in innate response to bacterial LPS, and critically contribute to periodontal pathogenesis [10]. Further investigation confirms that a strong interplay of LBP and cytokines is closely associated with periodontal conditions [11,12]. Taken together, these studies indicate that LBP expression in gingiva instantly acts on bacterial challenge and greatly accounts for periodontal homeostasis.

*Porphyromonas gingivalis* as a keystone periodontal pathogen may cause the shift of probiotic biofilms to pathogenic ones, and thereby result in the initiation and development of periodontal disease [13]. *P*. *gingivalis* LPS is one of the key virulence factors that is significantly involved in periodontal pathogenesis [14]. Interestingly, *P*. *gingivalis* could express two featured isoforms of LPS (penta-acylated LPS_1690_ and tetra-acylated LPS_1435/1449_) through alteration of lipid A structures under different micro-environmental conditions such as hemin levels and culture temperatures [15,16]. It has been shown that *P*. *gingivalis* LPS_1690_ and LPS_1435/1449_ differentially modulate innate host response, e.g. the expression of human β-defensin-2, pro-inflammatory cytokines and E-selectin [17–19]. Our recent studies further indicate that *P*. *gingivalis* LPS_1690_ could stimulate LBP expression in human oral keratinocytes (HOKs) through NF-κB and p38 MAPK signaling pathways, while *P*. *gingivalis* LPS_1435/1449_ is unable to do so [20, 21]. These findings collectively show that the shift of *P*. *gingivalis* LPS isoforms could crucially account for periodontal pathogenesis through disrupting the activities of innate defense molecules like LBP [22], but the underlying mechanisms need further investigation.

LBP is one of the important sensing apparatuses for Gram-negative bacterial LPS [4,22]. After binding to LBP in serum, LPS is transported to the TLR4/MD-2 signaling complex via soluble or membrane-anchored cluster of differentiation 14 (CD14), thereby triggering a cascade of pro- and anti-inflammatory responses [22]. LBP could sensitize or neutralize host cells to LPS stimulation at different concentrations [3]. While it remains unknown whether LBP could differentially interact with LPS lipid A structure and modulate host response. In the present study, we investigated the effects of LBP and its interactions with *P*. *gingivalis* LPS_1690_ and LPS_1435/1449_ on the expression of pro-inflammatory cytokines in HOKs, as well as the involvement of TLR signaling pathways. Interestingly, we found that LBP *per se* enabled to markedly up-regulate the expression of IL-6 and IL-8, and different isoforms of *P*. *gingivalis* LPS could interact differently with LBP and down-regulate to a great extent LBP-induced cytokine expression, likely through fine-tuning of the activities of CD180–MD1 complex and relevant TLRs.

## Materials and methods

### Cell culture

The primary HOKs were obtained from ScienCell Research Laboratories (Carlsbad, USA), and they were used in our recent study [20,21]. Cells were incubated in a serum-free oral keratinocyte medium (OKM) containing basal medium, 1% of growth factor supplement to HOKs and 1% of streptomycin and penicillin solution at 37°C with 5% CO_2_. The OKM was replaced every other day until the cells reached around 50% confluent, and it was then replaced daily. Cells at 3rd or 4th passages were subsequently employed in the experiments.

### Preparation of *P*. *gingivalis* LPS and LBP, and their interactions with HOKs

*P*. *gingivalis* (ATCC 33277) LPS was prepared by a well-established protocol via digesting cell extracts with proteinase K, and successive solubilization and precipitation [20,23,24]. The LPS was then purified by removal of endotoxin protein. The fraction of fatty acids was assessed by gas chromatography-mass spectroscopy, and tetra- (LPS_1435/1449_) and penta-acylated (LPS_1690_) lipid A structures were analyzed by the matrix-assisted laser desorption ionization time-of-flight mass spectrometry [20]. According to our previous studies [20,21], *P*. *gingivalis* LPS_1690_ (100ng/ml) and LPS_1435/1449_ (100ng/ml) were selected for the subsequent experiments. Recombinant human LBP (rhLBP) was obtained from the R & D Systems, Inc. (Minneapolis, MN, USA), and it was tested to have a purity over 97% by SDS-PAGE with the endotoxin level less than 0.10 EU per 1 μg of the protein by the limulus amebocyte lysate assay. Cells were pre-incubated with rhLBP at 10ng/ml, 100ng/ml and 1μg/ml for 1 h, prior to incubation with 100ng/ml of *P*. *gingivalis* LPS_1690_ or LPS_1435/1449_ for assessing the expression of mRNAs at 3 h and proteins at 24 h, respectively. Cells incubated with culture medium alone served as the negative controls.

### Reverse transcription and real-time PCR

Total cellular RNA was extracted from HOKs by RNeasy Mini Kit (Qiagen, Hilden, Germany), and 1 mg of RNA from each sample was reverse transcribed into cDNA in a final volume of 20μl with the QuantiTect® Reverse Transcription kit (Qiagen). RT-PCR was undertaken with the StepOne RT-PCR System (Applied Biosystems, Foster City, CA, USA), and β-actin served as the internal control. The primer sequences were as follows: IL-6 (NM_000600.3), forward: 5’-AATCATCACTGGTCTTTTGGAG-3’; reverse: 5’-GCATTTGTGGTTGGGTCA-3’; IL-8 (NM_000584.3), forward: 5’-GACATACTCCAAACCTTTCCACC-3’; reverse: 5’-AACTTCTCCACAACCCTCTGC-3’, and β-Actin (NM_001101.3), forward: 5’-TTG GCA ATG AGC GGT T-3’; reverse: 5’- AGT TGA AGG TAG TTT CGT GGA T-3’. The reaction conditions were set at 95°C for 20s, followed by 40 cycles at 95°C for 3s and 60°C for 30s. The data were analyzed by the comparative cycle threshold (Ct) method [25].

### Enzyme-linked immunosorbent assay (ELISA)

The levels of IL-6 and IL-8 in culture media were analyzed by ELISA (R & D Systems). The absorbance values were measured by a microplate reader (Victor, Vienna, VA, USA) at an optical absorbance of 450nm. The final concentrations were calculated with reference to the standard curves for IL-6 and IL-8, respectively.

### Blocking assay

The efficiency and specificity of anti-human TLR2 and TLR4 monoclonal antibodies (mAbs) (eBioscienceInc., San Diego, CA, USA) were tested as reported in our recent study [20]. Pam3Cys as a synthetic lipopeptide and TLR2 agonist was used to determine the blocking efficiency of anti-TLR2 mAb. Meanwhile, *E*. *coli* LPS as a representative TLR4 agonist was employed as the positive controls for the efficiency of anti-TLR4 mAb. HOKs were pre-incubated for 1 h with anti-TLR2 or anti-TLR4 mAbs at 10μg/ml, prior to incubation with 100ng/ml of Pam3Cys and *E*. *coli* LPS for 4 h (mRNAs) and 25 h (protein), respectively. IL-6 as a marker involved in TLR signaling pathways served as the outcome measure. Both anti-TLR2 and anti-TLR4 mAbs at 10μg/ml significantly blocked Pam3Cys- and *E*. *coli* LPS-induced IL-6 expression, respectively. The transcript and protein levels of IL-6 and IL-8 were detected by RT-PCR and ELISA, respectively. The cells incubated with OKM alone was used as the negative control, and those incubated with rhLBP and IgG served as the positive controls.

### Assay of human TLR signaling pathway

Total cellular RNA was reverse transcribed to cDNA by the RT^2^ First Strand Kit (SABiosciences, Frederick, MD, USA). RT-PCR and data analysis were performed with the standard protocol of RT^2^ Profiler^TM^ PCR Array System Human Toll-Like Receptor Signaling Pathway (SABiosciences). Briefly, cDNA templates were mixed with the RT^2^ qPCR master mix and H_2_O to form a 25μl cocktail. The cocktails were then aliquoted into each well coated with 96 different primers. The RT-PCR reaction conditions were set at 95°C for 10 min, followed by 40 cycles at 95°C for 15 s and 60°C for 1 min. Data analysis was performed using the SABiosciences web-based software for PCR array.

### Statistical analysis

Each experiment was performed in three replicates for each of three independent assays, and the data were presented as mean±SD. When both the normality test and test of homogeneity of variances were justified, the significant difference was analyzed by one-way ANOVA (Dunnett or Bonferroni or LSD test), otherwise by non-parametric methods. A *p*-value less than 0.05 was determined to be statistically significant. The datasets were analyzed by the IBM SPSS Statistic 19 (SPSS Inc, IBM Corp., Armonk, NY, USA).

## Results

### rhLBP and its interactions with *P*. *gingivalis* LPS modulated IL-6 and IL-8 expression

**Fig 1:**
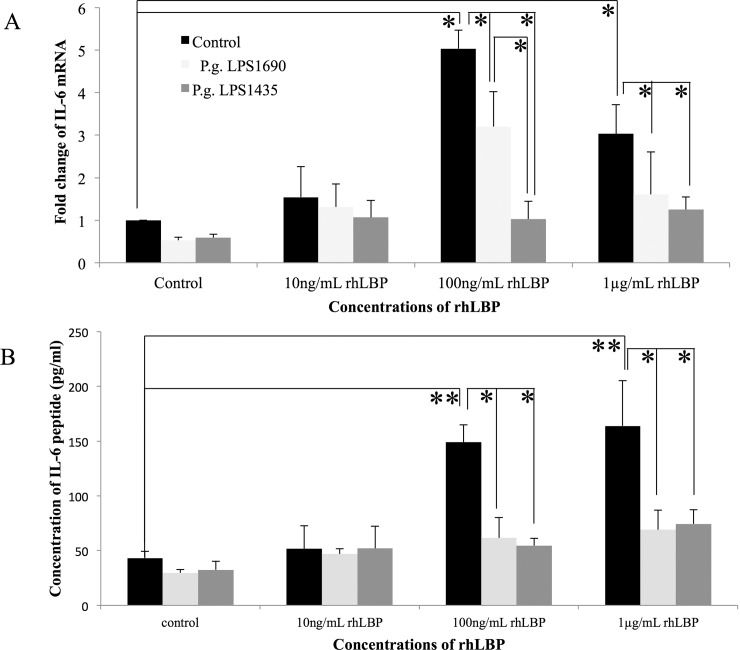
rhLBP and its interactions with *P*. *gingivalis* LPS_1690_ and LPS_1435/1449_ modulated the expression of IL-6 mRNA (A) and protein (B) in HOKs. rhLBP *per se* at 100ng/ml markedly up-regulated the transcript expression of IL-6 by around 5 folds at 3 h (A) (*p*<0.05), as well as its protein expression by around 3 folds at 24 h (B) (*p*<0.001). Notably, LPS_1435/1449_ down-regulated rhLBP (100ng/ml)-induced IL-6 more significantly than LPS_1690_ (around 80% vs. 40%, *p*<0.05) (A). One representative experiment of three is presented. *p < 0.05, **p < 0.001.

**Fig 2:**
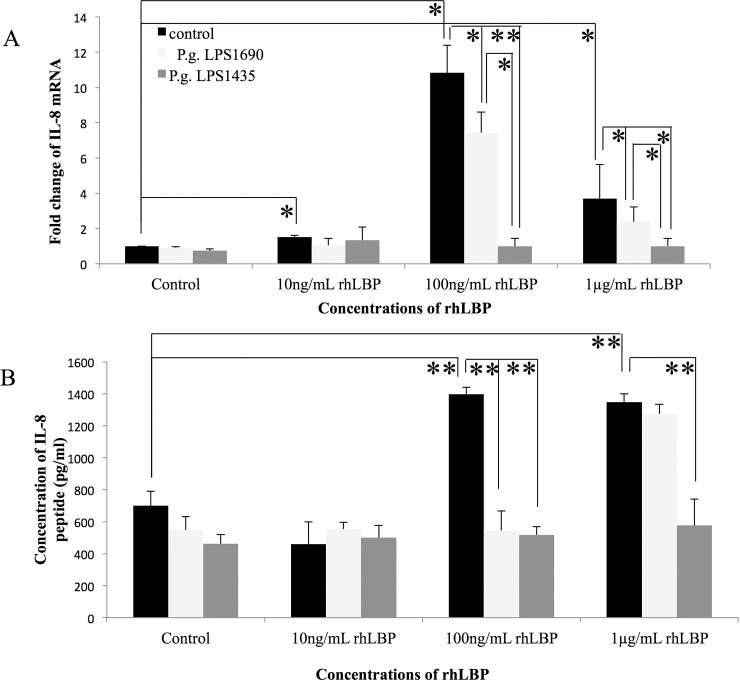
rhLBP and its interactions with *P*. *gingivalis* LPS_1690_ and LPS_1435/1449_ modulated the expression of IL-8 mRNA (A) and protein (B) in HOKs. rhLBP *per se* at 100ng/ml markedly up-regulated the transcript expression of IL-8 by around 11 folds at 3 h (A) (*p*<0.05), as well as its protein expression by around 2 folds at 24 h (B) (*p*<0.001). Notably, LPS_1435/1449_ down-regulated rhLBP (100ng/ml)-induced IL-8 mRNAs more significantly than LPS_1690_ (around 90% vs. 36%, *p*<0.001) (A). LPS_1435/1449_ down-regulated rhLBP (1μg/ml)-induced IL-8 proteins more significantly than LPS_1690_ (B). One representative experiment of three is presented. *p < 0.05, **p < 0.001.

It was worthy to note that rhLBP *per se* at 100ng/ml markedly up-regulated the transcript expression of IL-6 and IL-8 in HOKs at 3 h (Figs 1A and 2A) (*p*<0.05), as well as their protein expression at 24 h (Figs 1B and 2B) (*p*<0.001), respectively. Overall, the interaction of rhLBP with *P*. *gingivalis* LPS_1690_ or LPS_1435/1449_ down-regulated to different extents IL-6 and IL-8 expression, with reference to the controls. Notably, *P*. *gingivalis* LPS_1435/1449_ down-regulated rhLBP (100ng/ml)-induced IL-6 and IL-8 mRNAs more significantly than LPS_1690_ did (around 80% vs. 40%, *p*<0.05; and 90% vs. 36%, *p*<0.001, respectively) (Figs 1A and 2A). Moreover, LPS_1435/1449_ greatly down-regulated rhLBP-induced expression of IL-6 and IL-8 proteins, with reference to the controls (*p*<0.05) (Figs 1B and 2B).

### TLR2 antibody neutralized rhLBP up-regulated expression of IL-6 and IL-8

**Fig 3:**
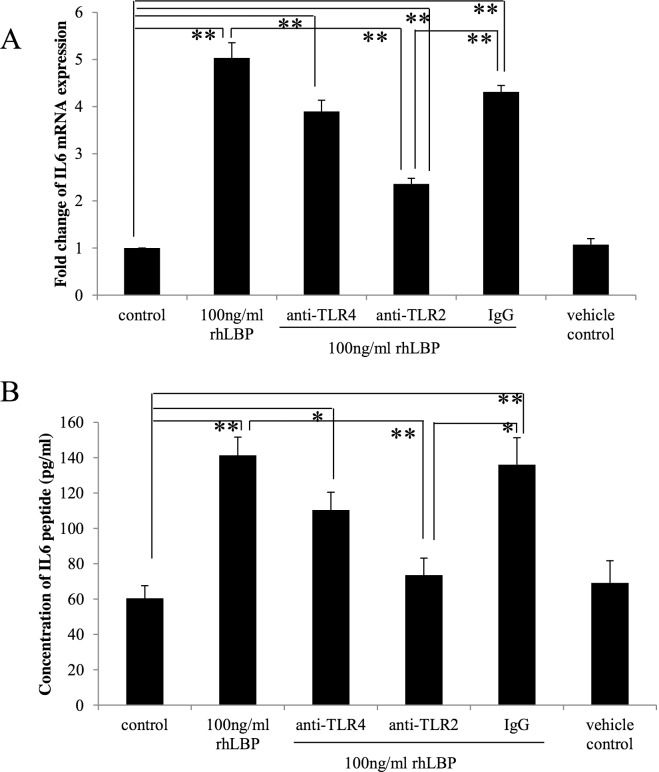
TLR2 antibody neutralized the modulatory effects of rhLBP on IL-6 expression in HOKs. Pre-incubation with TLR2 mAb (10μg/ml) significantly blocked rhLBP-induced expression of IL-6 mRNA at 4 h (A) and its protein at 25 h (B). However, both IL-6 mRNA and protein expression could not be significantly blocked by TLR4 mAb. One representative experiment of three is presented. *p < 0.05, **p < 0.001.

**Fig 4:**
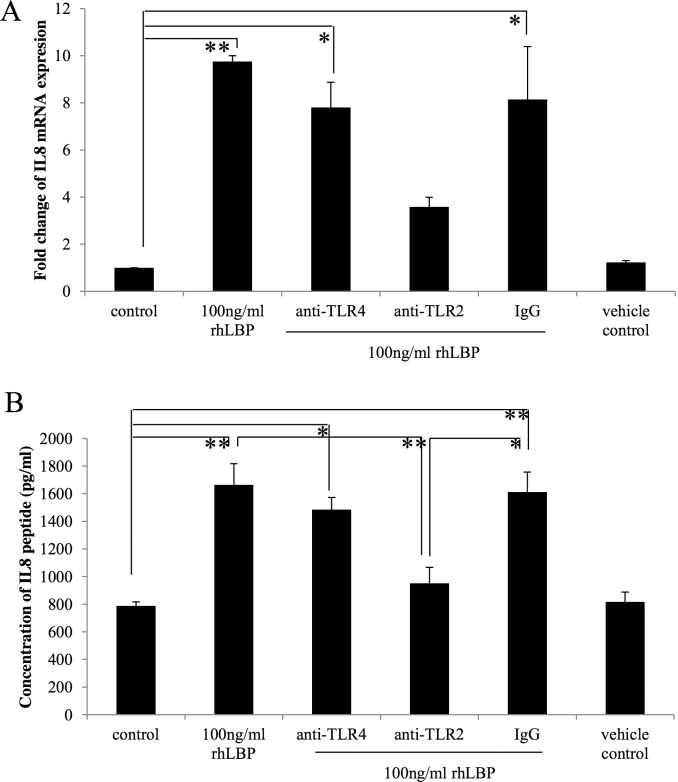
TLR2 antibody neutralized the modulatory effects of rhLBP on IL-8 expression in HOKs. IL-8 mRNA expression was down-regulated about 3 folds after TLR2 neutralization by TLR2 mAb at 10μg/ml, although there was no significant difference (A). Whereas, pre-incubation with TLR2 mAb (10μg/ml) significantly blocked rhLBP-induced expression of IL-8 protein at 25 h (B). However, both IL-8 mRNA and protein expression could not be significantly blocked by TLR4 mAb. One representative experiment of three is presented. *p < 0.05, **p < 0.001.

Next, to clarify whether TLR2 or TLR4 was involved in rhLBP-induced IL-6 and IL-8 expression in HOKs, blocking assays were undertaken. Pre-incubation with TLR2 mAb markedly blocked rhLBP-induced expression of IL-6 mRNA at 4 h (Fig 3A) as well as IL-6 (Fig 3B) and IL-8 proteins (Fig 4B) at 25 h (*p*<0.001). However, both IL-6 and IL-8 expression could not be significantly blocked by TLR4 mAb (Figs 3 and 4).

### rhLBP and its interactions with *P*. *gingivalis* LPS modulated the expression of genes associated with TLR signaling

A cluster of 89 genes involved in TLR signal transduction was analyzed simultaneously. HOKs were pretreated with rhLBP at 100ng/ml for 1 h, prior to incubation with 100ng/ml of *P*. *gingivalis* LPS_1690_ or LPS_1435/1449_ for 3h. As shown in Table 1, the downstream pathways of NF-ĸB, JNK/p38 and IRF were greatly involved in rhLBP-induced cytokine expression. Notably, the key mediators of TLRs and their adaptors such as CD180 (a TLR4 antagonist) and MD-1 (a MD-2 homolog) were significantly down-regulated by rhLBP *per se*. Both CD180 and MD-1 were dramatically up-regulated by *P*. *gingivalis* LPS_1435/1449_, whereas these two genes were reversely modulated by *P*. *gingivalis* LPS_1690_ (20.86 folds and -6.93 folds, respectively).

**Table 1:** The changes of gene expression associated with the Toll-like receptor signaling in the three test groups of HOKs with reference to the control cells. The genes up-regulated over 2 folds are highlighted in bold, and those down-regulated over 2 folds are marked in bold italics.

Functions	Gene products	Fold change with reference to the control group		
		rhLBP	rhLBP + *P*. *gingivalis* LPS_1690_	rhLBP + *P*. *gingivalis* LPS_1435/1449_
Toll-like receptors	CD180	***-2*.*44***	**20.86**	**7.11**
	TLR1	-1.24	-1.56	***-2*.*07***
	TLR8	1.14	1.59	**4.04**
	TLR9	-1.61	1.09	***-2*.*18***
	TLR10	1.91	-1.66	***-4*.*65***
Adaptors & TLR interacting protein	BTK	1.12	**3.40**	**3.97**
	MD-1	***-9*.*62***	***-6*.*93***	**4.05**
NF-ĸB pathway	CSF2	**2.33**	**3.63**	1.28
	CSF3	1.72	**2.05**	1.08
	INFB1	**9.15**	**8.89**	0.77
	IFNG	1.14	1.59	**4.05**
	IL2	1.14	1.59	**19.15**
	IL-6	**9**	**2.49**	0.86
	IL-8	**13.19**	**6.00**	1.13
	IL10	0.98	**2.59**	**4.39**
** **	LTA	1.21	**2.09**	0.69
JNK/p38 pathway	FOS	**2.04**	1.98	1.15
NF/IL-6 pathway	CLEC4E	1.14	**14.07**	**12.36**
** **	PTGS2	**2.68**	**2.78**	0.63
IRF pathway	CXCL10	**2.1**	**2.13**	1.22
** **	IFNB1	**9.15**	**8.89**	1.14
** **	IFNG	1.14	1.59	**4.05**
Regulation of adaptive immunity	CD86	-1.95	***-5*.*18***	***-4*.*57***

## Discussion

LBP as an acute phase protein plays an important role in modulation of immuno-inflammatory response to bacterial LPS [4]. It functions as a shuttle protein to remove the monomer of LPS molecules from LPS aggregates, transporting them to cell surfaces and then getting back to the aggregates [2]. Moreover, LBP is not only present as a soluble form but also exists in a membrane-bound form (mLBP) that intercalates into cell membrane. It acts as a fusion protein for LPS aggregates to lipid membranes [26–29]. However, the other functions of LBP remain to be investigated. Classical studies have shown that the dual concentration-dependent effects of LBP in its interaction with LPS act through different approaches, i.e. enhancement of bacteria-induced cellular activation at a low concentration through mLBP intercalation, and neutralization of the LPS effects at a high concentration through soluble LBP [3, 27]. Although the active interactions of LBP with LPS or other bacterial components have been well documented, it is considered that LBP alone is unable to induce detectable expression of TNF mRNA and protein [2]. Interestingly, our present study reveals that rhLBP *per se* enables to significantly modulate the expression of pro-inflammatory cytokines and related TLR signaling molecules in HOKs with reference to the negative controls. This observation could be elaborated from different lines. Firstly, the discovery of LBP as a transmembrane protein makes it structurally possible to induce signaling pathway without necessary interaction with LPS [28]. The soluble rhLBP added in the culture media may intercalate directly into the cell membrane, thereby interacting with other cytoplasmic receptors and subsequently inducing cell activation. Secondly, LBP appears to be able to bind not only to LPS, but also to other bacterial compounds including viable bacteria *per se* and other surface patterns such as lipoproteins, lipoteichoic acid (LTA) and glycolipids [30]. According to the manufacturer, the rhLBP used in the present study has been tested with its purity above 97% and endotoxin level below 0.10 EU/1 μg of the protein. Although it is unlikely that the interaction of rhLBP with other ligands induces notable cellular response, such a possibility could not be entirely excluded. Furthermore, alternative protein control such as heat-denatured LBP protein could have been added for additional reference other than the negative control used. Further study is required to clarify the current findings and underlying mechanisms.

TLR2 and TLR4 are strongly involved in the expression of immuno-inflammatory molecules. TLR2 could dimerize with other TLRs for recognition of a diversity of structurally different ligands from bacteria and fungi. The TLR2 ligands include triacylated lipopeptides like Pam3CSK4 that interacts with TLR1/TLR2, and diacylated lipid/lipopeptides like LTA and *Mycoplasma fermentan* interacting with TLR2/TLR6 complex [31]. TLR4 is mainly involved in LPS-induced signaling [32]. In the present study, rhLBP up-regulated the expression of IL-6 and IL8 could be greatly down-regulated by anti-TLR2 mAb but not anti-TLR4 mAb, suggesting that TLR2 signaling may critically account for rhLBP-induced expression of pro-inflammatory cytokines.

It has recently been shown that *P*. *gingivalis* LPS may be shifted from its penta-acylated LPS_1690_ isoform to the tetra-acylated LPS_1435/1449_ isoform in high hemin levels and increased culture temperature [15,16]. Our previous studies have shown that the expression of pro-inflammatory mediators and antimicrobial proteins could be significantly dysregulated by *P*. *gingivalis* LPS_1435/1449_, and the innate host defense may therefore be weakened, thereby contributing to periodontal pathogenesis [17,18, 20–22]. As shown in the present study, rhLBP *per se* can act on host cells to promote the expression of pro-inflammatory cytokines and modulate immuno-inflammatory response. As *P*. *gingivalis* LPS_1435/1449_ could more significantly down-regulate rhLBP-induced expression of IL-6 and IL-8 with reference to *P*. *gingivalis* LPS_1690_, rhLBP could function as a potential innate immune enhancer and contribute to periodontal homeostasis. Its possible clinical uses and relevant implications are warranted for further investigation. In addition, currently it remains unknown how and to what extents host cells may respond to different phenotypes of *P*. *gingivalis* cells with heterogeneous lipid A structures of LPS under various microenvironmental conditions. Hopefully, our ongoing study on the modulatory effects of various phenotypes of *P*. *gingivalis* cells on the expression profiles of innate host defense molecules could shed some light on this important issue.

Our present study suggests that the downstream pathways of NF-ĸB, JNK/p38 and IRF may be involved in rhLBP-enhanced cytokine expression. Notably, the key mediators of TLRs and their adaptive proteins such as CD180 and MD-1 could be significantly down-regulated by rhLBP. Moreover, both CD180 and MD-1 mRNAs were dramatically up-regulated by *P*. *gingivalis* LPS_1435/1449_, while these transcripts were reversely modulated by *P*. *gingivalis* LPS_1690_ (20.86 folds and -6.93 folds, respectively). The MD-2/TLR4-dependent host response to bacterial LPS is related to the complex of MD-1 and TLR homolog radioprotective 105 (RP105, also termed as CD180) [33]. CD180 as a type I transmembrane protein shares about 30% sequence identity with TLR4, and MD-1 sharing around 20% similarity with MD-2 belongs to the same family of MD-2 with a defined lipid binding function [34,35]. MD-1 has been identified as a molecule strongly associated with CD180 [36]. Indeed, CD180/MD-1 as a cell surface molecular complex in the TLR family crucially controls LPS-induced downstream signaling [37]. Besides the common features of CD180/MD-1 and MD-2/TLR4, they are also functionally interrelated. It has been confirmed that the LPS action can be inhibited by CD180/MD-1 through interacting with MD-2/TLR4 in HEK293 cells and immune cells such as dendritic cells and macrophages [38]. In B-cells, the CD180/MD-1 complex could impair the production of TLR-dependent antibody against LPS or lipoproteins [33,39].

Based on the above findings, the underlying mechanism by which the two isoforms of *P*. *gingivalis* LPS exert different effects on rhLBP-induced expression of pro-inflammatory cytokines may be postulated. Herein, rhLBP could interact with the HOKs through various possible pathways. Firstly, as concerned above it may directly interact with other cytoplasmic receptors and activate cells. Secondly, it could bind to the penta-acylated isoform for inducing the TLRs signaling pathway, and/or to the tetra-acylated isoform for partially blocking the signaling concerned. Moreover, the penta-acylated lipid A structures are TLR4 agonists, and the tetra-acylated ones are antagonists to TLR4 [19, 24, 40]. Our study further shows that CD180 and MD-1, the key mediators of TLRs and their adaptors, can be significantly down-regulated by rhLBP; while they could be greatly up-regulated by *P*. *gingivalis* LPS_1435/1449._ Thus, these observations collectively suggest that rhLBP may possibly modulate to different extents the expression of IL-6 and IL-8 induced by the different isoforms of *P*. *gingivalis* LPS, through regulation of the activity of CD180-MD1 complex that acts as an antagonist of TLR4-MD-2 complex. Further study is required to clarify the mechanisms underlying LBP modulation of cytokine expression in HOKs.

The present study shows that rhLBP *per se* enables to significantly up-regulate the expression of pro-inflammatory cytokines in HOKs through TLR2 signaling pathway. *P*. *gingivalis* LPS with different lipid A structures down-regulates to different extents rhLBP-induced cytokine expression, possibly through fine-tuning of the CD180-MD1 complex and relevant TLRs. The current findings may enhance our understanding of the innate defense mechanisms in periodontal homeostasis. This study could contribute to the development of novel immuno-modulators for prevention and control of common inflammatory diseases like periodontitis.
