# Correction: 3LPS-binding protein and its interactions with *P. gingivalis* LPS modulate pro-inflammatory response and Toll-like receptor signaling in human oral keratinocytes

**DOI:** 10.1371/journal.pone.0176996

**Published:** 2017-05-01

**Authors:** The PLOS ONE Staff

The number “3” should be omitted from the article title. The publisher apologizes for the error.

The correct title is: LPS-binding protein and its interactions with *P*. *gingivalis* LPS modulate pro-inflammatory response and Toll-like receptor signaling in human oral keratinocytes.

The correct citation is: Ding P-H, Darveau RP, Wang C-Y, Jin L (2017) LPS-binding protein and its interactions with *P*. *gingivalis* LPS modulate pro-inflammatory response and Toll-like receptor signaling in human oral keratinocytes. PLoS ONE 12(4): e0173223. https://doi.org/10.1371/journal.pone.0173223
